# The Effect of Phenol Composition on the Sensory Profile of Smoke Affected Wines

**DOI:** 10.3390/molecules20069536

**Published:** 2015-05-26

**Authors:** David Kelly, Ayalsew Zerihun

**Affiliations:** Department of Environment and Agriculture, Margaret River Education Campus, Curtin University, Margaret River, WA 6285, Australia; E-Mail: A.Zerihun@curtin.edu.au

**Keywords:** smoke taint, wine, volatile phenols, glycoconjugated phenols, sensory analysis

## Abstract

Vineyards exposed to wildfire generated smoke can produce wines with elevated levels of lignin derived phenols that have acrid, metallic and smoky aromas and flavour attributes. While a large number of phenols are present in smoke affected wines, the effect of smoke vegetation source on the sensory descriptors has not been reported. Here we report on a descriptive sensory analysis of wines made from grapes exposed to different vegetation sources of smoke to examine: (1) the effect vegetation source has on wine sensory attribute ratings and; (2) associations between volatile and glycoconjugated phenol composition and sensory attributes. Sensory attribute ratings were determined by a trained sensory panel and phenol concentrations determined by gas chromatography-mass spectroscopy. Analysis of variance, principal component analysis and partial least squares regressions were used to evaluate the interrelationships between the phenol composition and sensory attributes. The results showed that vegetation source of smoke significantly affected sensory attribute intensity, especially the taste descriptors. Differences in aroma and taste from smoke exposure were not limited to an elevation in a range of detractive descriptors but also a masking of positive fruit descriptors. Sensory differences due to vegetation type were driven by phenol composition and concentration. In particular, the glycoconjugates of 4-hydroxy-3-methoxybenzaldehyde (vanillin), 1-(4-hydroxy-3-methoxyphenyl)ethanone (acetovanillone), 4-hydroxy-3,5-dimethoxybenzaldehyde (syringaldehyde) and 1-(4-hydroxy-3,5-dimethoxyphenyl)ethanone (acetosyringone) concentrations were influential in separating the vegetation sources of smoke. It is concluded that the detractive aroma attributes of smoke affected wine, especially of smoke and ash, were associated with volatile phenols while the detractive flavour descriptors were correlated with glycoconjugated phenols.

## 1. Introduction

Wines produced from vineyards exposed to bushfire smoke often have unpalatable levels of smoke related attributes including smoky, metallic, bitter, ash and medicinal flavour and aroma sensory descriptors [1,2]. These detractive sensory descriptors in wine, known as smoke taint, have low consumer acceptance and are due to the uptake of smoke-borne lignin derived phenols into grapes [3], which remain in finished wines [4]. 

Our knowledge of smoke taint in grapes and wines has increased considerably in the last five years. For example, we now know that smoke affected wines contain many volatile and glycoconjugated phenols [3,4,5,6,7]. Our understanding of the sensory impacts is also evolving. The sensory profile of smoke affected wines is thought to be closely related to the volatile phenol composition; however it has been recently shown that glycoconjugated phenols may deconjugate to the volatile forms in the mouth during tasting [2] and may have direct impact on the flavour profile [8]. While each phenol has a characteristic sensory threshold in wine, a synergistic effect is expected to occur where a combination of phenols below their respective threshold concentrations have a significant effect on the sensory properties [9,10].

Kelly *et al.* [3] reported that fuel lignin makeup, and therefore bushfire source, was surprisingly not a good indicator of the types of lignin pyrolysis products that become elevated in wines. Building on this, in this study we have utilised the sensory descriptors of smoke taint [2] to investigate differences in the sensory profile due to bushfire smoke source. The associations of smoke taint descriptors with volatile and glycoconjugated phenol concentrations have also been examined to explore the phenol drivers for the harsh organoleptic properties that render smoke affected wines unacceptable to consumers. Previous studies have explored some of these associations [2,8] in commercial wines exhibiting smoke taint characteristics. However, in such studies, the commercial nature of the wine samples adds several sources of variation. These include: (a) wines being from a range of varieties, with likely differences in ethanol and acid profiles; and (b) wines aged in toasted oak barrels, a winemaking process that imparts smoky, toasted aromas and flavours by extracting oak lignin derived phenols into the wine. Accordingly, the aims of this study were to: (a) determine if the bushfire smoke source affects the sensory properties of the wines; and (b) investigate associations between the detractive sensory properties and a broader range of smoke-derived phenols than have been considered hitherto. To this end, wines have been evaluated by a trained sensory panel and the concentrations of volatile and glycoconjugated phenols determined where a single variety has been exposed to controlled, replicated smoke emissions of three main vegetation types found in the southwest of Western Australia [3]. The vineyard smoking trials exposed replicated panels of vines of similar vigour and at the same phenological stage to produce wines with similar ethanol, acid and residual sugar profiles. The experiment was therefore designed to allow comparisons of wines due to smoke vegetation type and phenol concentrations without confounding underlying differences in winemaking.

## 2. Results and Discussion

### 2.1. Results

The control wines and wines made from smoke exposed grapes had comparable basic wine chemistry attributes (pH, acidity, alcohol, and concentrations of residual sugars (glucose and fructose) and hydroxybutanedioic acid (malic acid)) and were free of winemaking faults as shown by low levels of volatile acidity (Table 1). 

**Table 1:** Standard wine chemistry measures. Data are mean ±1 standard error (*n* = 5). Total Acidity (TA) is grams per litre equivalent of 2,3-dihydroxybutanedioic acid (tartaric acid) and Volatile Acidity (VA) is grams per litre equivalent of acetic acid. Alcohol is weight per volume percentage of ethanol. Malic acid and residual sugars (sum of glucose and fructose) were below quantification limits (0.18 g/L and 0.1 g/L respectively).

Treatment	pH	TA	Alcohol	VA
Control	3.56 ± 0.03	4.7 ± 0.3	13.8 ± 0.1	0.304 ± 0.002
Karri	3.56 ± 0.01	4.6 ± 0.1	13.9 ± 0.1	0.300 ± 0.002
Oats	3.57 ± 0.02	4.7 ± 0.1	13.6 ± 0.1	0.298 ± 0.002
Pine	3.58 ± 0.02	4.4 ± 0.1	13.8 ± 0.1	0.300 ± 0.002

**Figure 1:**
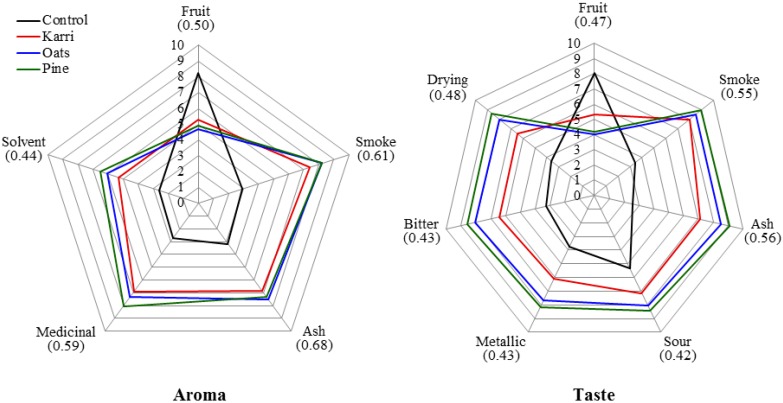
Influence of vegetation source of smoke on mean aroma (**left panel**) and taste (**right panel**) intensity ratings of Merlot wines. Numbers in parentheses following each descriptor are the least significant differences (*p* = 0.05).

The sensory scores (aroma and taste) of wines made from smoke treatment were significantly different in every attribute compared to the unsmoked control wines regardless of fuel type (Figure 1).

Smoke treatment significantly increased the detractive aroma and taste scores and decreased overall fruit aroma and taste scores compared to the control wines (Figure 1). Although significantly greater than the control wines in each non-fruity sensory descriptor of taste and aroma, the karri treatment wines were not significantly different in aroma scores from the oats and pine treatment wines (except solvent aroma where they were significantly less than the pine treatments, Figure 1). The karri treatment wines were significantly higher in fruit taste compared to the oats and pine treatment wines and significantly lower in each negative taste descriptor except smoke taste for which all three fuels had similar impact (Figure 1). Across each of the twelve sensory descriptors there was no significant difference in the oats and pine treatments.

**Figure 2:**
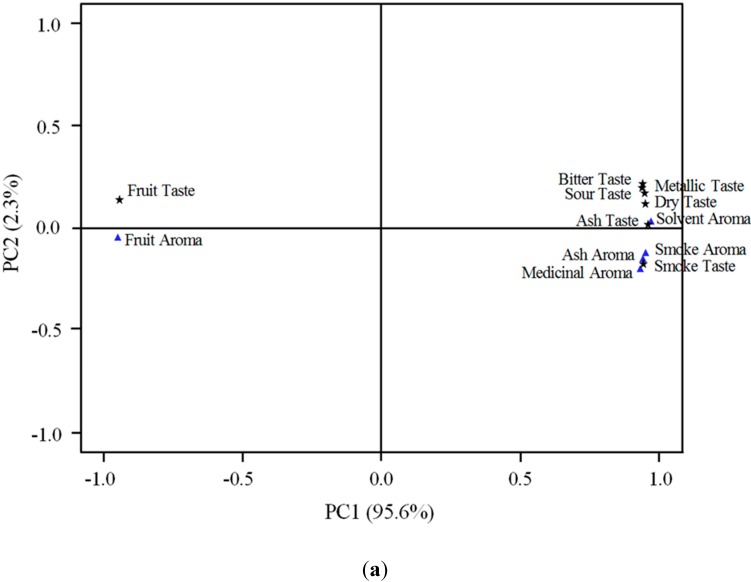

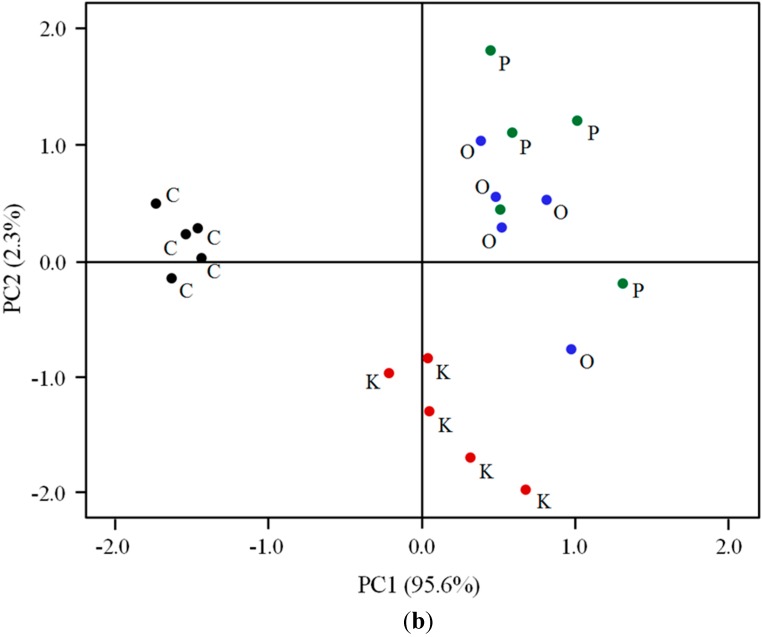
Differentiation of wines made from grapes exposed to smoke of different fuel types based on principal component analysis of sensory attributes: plots of (**a**) the sensory attributes vector loadings; and (**b**) the resultant scores of the replicates of fuel treatments shown on the first two principal axes of variation.

Principal components analysis (PCA) of the twelve sensory descriptors revealed that the variation embodied in the twelve sensory attributes could be effectively summarised by a single axis of variation (95.6% of total variance, *p* < 0.0001), representing a fruity-smoky sensory spectrum (Figure 2a,b). Correspondingly, the smoke treatment wines were segregated on fruit and non-fruit aroma and taste attributes. The control wines were characterised by high fruit taste and aroma and low smoke related sensory attributes. By contrast, the smoke treatment wines, particularly of oat and pine fuel, were strongly and similarly associated with low fruity characters and high smoke attributes (Figure 2a,b). The karri treatment wines, falling intermediate along the fruit-smoke sensory attributes spectrum, were distinct from both the control and the oats and pine treatments.

**Figure 3:**
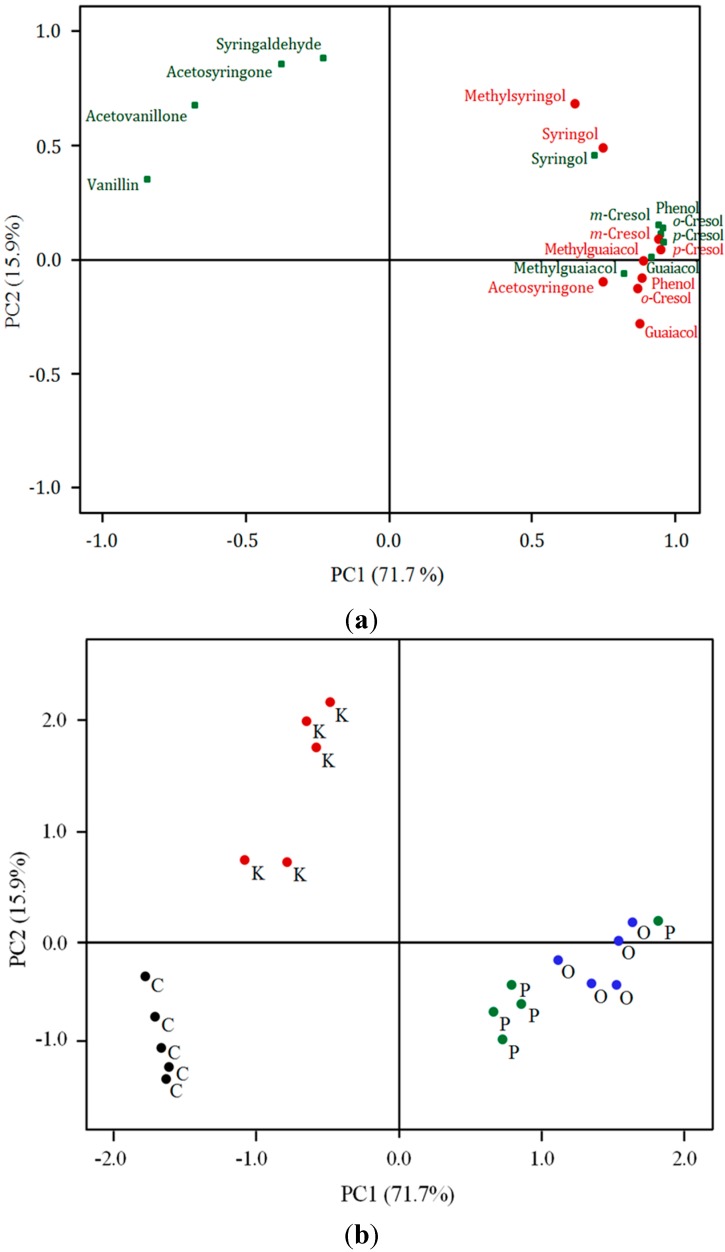
Separation of wines made from grapes exposed to smoke of different fuel types based on principal component analysis of wine volatile and glycoconjugated phenol composition: plots of (**a**) the vector loadings of the volatile (red circles) and glycoconjugated phenols (green squares); and (**b**) the resultant fuel treatment scores displayed on the first two principal axes of variation.

**Figure 4:**
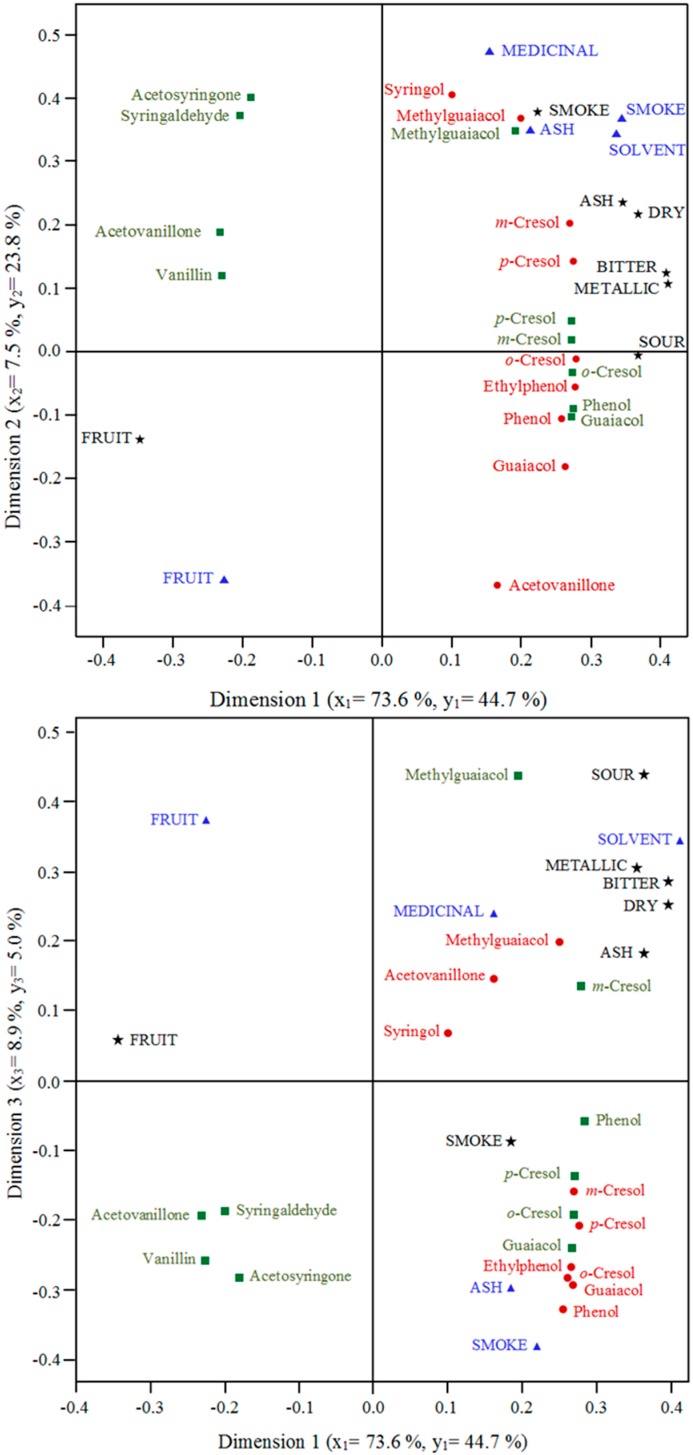
Correlation loading biplots (of loadings and scores) from partial least square regressions analysis of wine phenol composition and sensory attributes. Wine phenol composition (x_i_) with glycoconjugated phenols (green squares) and volatile phenols (red circles). Wine sensory attributes (y_i_) with aroma attributes (blue triangles) and taste attributes (black stars).

A principal component analysis of the volatile and glycoconjugated phenols showed the first and second components jointly extracted 87.6% of the total variance (Figure 3a,b). The first PC (~72%) was strongly and positively correlated with volatile and glycoconjugated phenols except the glycoconjugates of vanillin, acetovanillone, acetosyringone and syringaldehyde with which it was negatively associated (Figure 3a). As such, PC1 represented an index of intensity of “smokiness”. The second component, accounting for 16% of the variance, had moderate to strong positive associations with glycoconjugates of syringaldehyde, acetosyringone, acetovanillone, vanillin, and volatile 2,6-dimethoxy-4-methylphenol (4-methylsyringol) and 2,6-dimethoxyphenol (syringol) (Figure 3a). The resultant principal component scores of the fuel smoke treatments are displayed in Figure 3b. The first PC clearly differentiated the treatments based on overall smoke index, from low level in the controls to intermediate in the karri and high for the oat and pine fuel treatments. Along the second dimension, the fuel effects are further differentiated on the basis of their relative contents of glycoconjugates of acetosyringone, syringaldehyde, acetovanillone, vanillin, syringol and volatile syringol and 4-methylsyringol. Thus, the karri treatment wines (which had relatively high concentrations of acetosyringone, syringaldehyde, acetovanillone, vanillin, syringol and volatile syringol and 4-methylsyringol) were further differentiated from the pine and oat treatment wines. The relative proximity of the karri treatment wines to the control wines were driven by their similarity in the levels of the glycoconjugates of vanillin and acetovanillone (*cf*. Figure 3a,b). Correlation loading biplots from partial least squares regression analysis of volatile and glycoconjugated phenols and sensory descriptors are shown in Figure 4. These results indicate that generally the detractive aroma attributes were closely correlated with the volatile phenols whereas the taste descriptors appear to correlate with the glycoconjugated phenols.

### 2.2. Discussion

Merlot wines made from grapes exposed to smoke emissions from the three vegetation fuel types in this study were significantly higher in putative taint phenols than unsmoked control wines [3]. Karri, pine and oats treatment wines and unsmoked control wines have been evaluated here to determine the influence wine phenol composition has on the sensory attributes of smoke affected wine. In this work, smoke application and winemaking procedures had no significant effects on acidity, sugars, alcohol or volatile acidity in the wines (Table 1), indicating that smoke exposure impacts on sensory differences are not confounded by variations in the basic chemical profiles of the wines. Clear differences in aroma and taste (Figure 1) were found in wines from smoking applications. Earlier work [2] showed that bitterness, sour and solvent descriptors were not associated with ‘smoke sensory attributes’. In contrast, in the current work where the wines were made from fruit that had the same maturity and ferments were carried out under the same conditions (Table 1), these attributes, especially the solvent intensity rating, were correlated with smoke sensory descriptors (Figure 2a and Figure 4). This outcome suggests that when wine background differences in VA and TA (which were features of earlier studies [2]) are eliminated, as was the case in the current study, panelists detected higher solvent aroma intensity in smoke affected wines. However, further study is warranted to resolve these apparent, possibly matrix-related, discrepancies. The control wines were significantly higher in fruit aroma and taste and significantly lower in each of the negative sensory aroma and taste attributes compared to the karri, pine and oats treatment wines (Figure 1). Differences were also apparent between the *Eucalypt* karri treatment and the pine and oats treatment wines. While karri treatment wines were significantly different to the control wines in each aroma and taste attribute they also had significantly more fruit taste and were lower in each negative taste attribute compared to both pine and oats treatments, except for smoke taste where there was no difference (Figure 1). This was reflected by significantly lower glycoconjugated phenol concentrations in the karri treatment wines compared to the pine and oats treatment wines [3] as well as relatively higher acetovanillone, acetosyringone and syringaldehyde levels which confer soft and sweet undertones [11] and thus possibly partly mitigating the negative sensory impact of the other wine phenols. Additionally, the observation that aroma attributes tend to correlate with volatile phenols (Figure 4) may explain the comparable aroma profile of wines from the three fuel types since their volatile phenols compositions were of similar magnitude. Similarly, the association of taste descriptors with phenol glycoconjugates may underlie why the detractive taste descriptors were less pronounced in karri treatment wines than in pine or oat treatment wines. The sensory profile of the pine treatment wines were closely aligned to the oats treatments with no significant differences in any of the aroma or taste sensory scores. Although the lignin makeup of fuels was not found to be a good indicator of phenol composition in smoke affected wines [3], the karri treatment wines have distinct differences in taste profile (Figure 1) and phenol composition [3] from the oats and pine treatments. This is clearly seen in plots of the principal component scores of wine volatile and glycoconjugated phenols and sensory attributes in which the karri fuel treatment wines are separated from both the oats and pine treatments (*cf.* Figure 2b and Figure 3b). The drivers for separation in sensory profile in the karri treatment wines are a closer alignment to glycoconjugates of acetosyringone, syringaldehyde, vanillin and acetovanillone as well as the relatively low concentrations of the other glycoconjugates, as described earlier. Vanillin and acetovanillone are usually associated with extraction from oak barrels during wine aging [12] and may also form from transformation of (*E*)-3-(4-hydroxy-3-methoxy-phenyl)prop-2-enoic acid (ferulic acid) and 4-ethenyl-2-methoxyphenol (4-vinylguaiacol) by malo-lactic bacteria [13]. The winemaking here did not include contact with oak products. The apparent high levels of vanillin and acetovanillone in the control and karri treatment wines thus suggests that the high levels of phenols in the pine and oat treatment wines restricted bacterial transformation of ferulic acid and 4-vinylguaiacol to vanillin and acetovanillone by malo-lactic bacteria. This is worthy of further investigation. Vanillin and acetovanillone impart a powerful aroma and flavour characteristic of vanilla [13] and acetosyringone and syringaldehyde impart soft, sweet undertones [11] which in comparison to the other phenols examined here is considered a positive sensory attribute.

Toth and Potthast [14] describe phenol, the cresols, guaiacol and 4-methylguaiacol as having a hot, bitter taste and this is consistent with the pattern of association seen in Figure 4. The harsh ash, sour, metallic, bitter and drying tastes of smoke tainted wines [2] were closely associated and highly correlated with glycoconjugates of phenol, *m-*cresol and *p-*cresol in the smoke treatment wines (Figure 4) and these phenol glycoconjugates are therefore most likely the drivers for the harsh taste of smoke affected wines. Parker *et al.* [2] found *m*-cresol and guaiacol β-d-glucosides impart undesirable flavours in smoke affected wine by deconjugation in the mouth to release volatile phenols. A large number of glycoconjugated phenols were associated with negative smoke taint flavour descriptors in this study. This supports the findings of Parker *et al.* [2] and suggests several phenols and their glycoconjugates may also contribute to the complex palate of taste descriptors described in this sensory assessment. While the amelioration of wine by reverse osmosis [15] may remove the sensory influence of volatile phenols, the negative taste descriptors associated with the phenol glycoconjugates are expected to remain unchanged.

Earlier work has focussed on guaiacol concentration as an indicator of smoke taint in wine [16,17,18] with smoke aroma and taste descriptors reportedly due to this compounds volatile concentration [2,19]. In this study, 4-methylguaiacol was found to have a closer association than guaiacol to solvent aroma, ash and dry taste despite volatile guaiacol concentrations being up to four times the 4-methylguaiacol concentrations in smoke treatment wines. Wasserman [20] calculated the flavour and aroma index of 4-methylguaiacol in water as being significantly higher (~13 times) than guaiacol and the partial least square regression (Figure 4) shows volatile 4-methylguaiacol and not guaiacol to be closely aligned to both smoke aroma and taste. Interestingly, the karri treatment wines were significantly less in both volatile and glycoconjugates of guaiacol and 4-methylguaiacol (Tables 5 and 6 in [3]) than the oats and pine treatment wines and yet there were no significant differences in the descriptors of smoky aroma or smoky flavour between the three treatments. Syringol also contributes to smoke aroma and taste [20] but has a lower taste and odour index than guaiacol (~1/4) and although syringol concentrations in all smoking treatments were well below aroma and taste thresholds (1.85 and 1.65 ppm respectively, [20]) the synergistic influence of syringol may be important in contributing to smoke taste and aroma.

Although earlier work has focussed on volatile guaiacol concentrations as an indicator of smoke taint in wine [16,17,18], this study has found the glycoconjugates of phenol, *m-*cresol and *p-*cresol more closely aligned to the harsh smoke taint descriptors [2] associated with taint in wine made from smoke exposed grapes.

## 3. Experimental Section

Wines for sensory analysis were made from the fruit of vines that were experimentally exposed to smoke as described in Kelly *et al.* [3].

### 3.1. Vineyard Smoking Trials

Vine smoking experiments were conducted in the southwest of Australia in the Margaret River viticultural region (33°57ʹS, 115°01ʹE) as described in previous works [3,4]. Vegetation fuels that typically contribute to summer fire events were chosen for the study and compiled from component proportions that burn in ten year old fuel accumulation [3,21]. Three fuels; a hardwood forest species, karri (*Eucalyptus diversicolor* F. Muell.), a softwood plantation species, pine (*Pinus radiata* D. Don.) and a local pasture species, wild oats (*Avena fatua* L.), from the previous work [3] were chosen for the sensory study. Smoke exposures of ten year old *Vitis vinifera* L. cv. Merlot vines were set up as a completely randomised block design with panels of vines selected for equal vigour and crop load. For each vegetation fuel type, smoke treatments of five replicate panels were randomly allocated with control replicate panels having no smoke exposure (a total of 20 experimental units). In each smoke exposure replicate, a kilogram of fuel was pyrolysed at wildfire temperatures [22] in a purpose built incinerator [3] and the smoke trapped over the vines for 30 min in a 63 m^2^ tent. Smoking treatments were applied 14 days post veraison (EL 36). The compilation of fuels, fuel lignin analysis, pyrolysis, emissions analysis and vineyard trials are described in detail in Kelly *et al.* [3].

### 3.2. Winemaking

Six weeks after smoking application, the fruit from each replicate was harvested separately at commercial maturity (~23% total soluble solids). Wines were made individually by standard red winemaking methods [3] where the fruit was crushed and de-stemmed with the addition of 100 mg/L potassium metabisulphite and total acids adjusted to 7.0 g/L with additions of tartaric acid. The musts were fermented on skins to dryness (<1 g/L residual sugars) in glass demijohns with the addition of 300 mg/L *Saccharomyces cerevisiae* EC1118 (Lallemand Inc., Montreal, QC, Canada) and 100 mg/L diammonium hydrogen phosphate added as a nitrogen supplement. Upon completion of fermentation, the musts were pressed off skins, racked from gross lees and inoculated with 10 mg/L *Oenococcus oeni* (Viniflora CH 16, CHR Hansen, Hørsholm, Denmark) to initiate malolactic conversion. When less than 0.1 g/L malic acid remained, the wines were sulphured with 60 mg/L potassium metabisulphite, cold stabilised at −4 °C for 21 days, filtered through a 0.2 µm pore size cartridge (Sartorius Sartopure 2 Maxicap, Sartorius, Gottingen, Germany) and bottled under stelvin closure.

### 3.3. Wine Analysis

Volatile and glycoside bound lignin derived phenols were analysed using gas chromatography—mass spectrometry as described by Singh *et al.* [7] and results reported for the unsmoked control and the three treatment fuels of karri, pine and wild oats in Kelly *et al.* [3]. The wines were also analysed by Fourier Transform Infra-Red Spectroscopy (Oeno Foss Type 4101, Foss, Hillerød, Denmark) for alcohol (% w/v), residual sugars (sum of glucose and fructose), malic acid concentration (g/L) and volatile acidity (acetic acid g/L equivalent). Titratable acid (tartaric acid g/L equivalent) and pH were determined as described by Iland *et al.* [23].

### 3.4. Sensory Analysis

Sensory assessments of wines were conducted in a well ventilated room purpose built for wine sensory analysis. Sixteen respondents (eight female and eight male, 25 to 55 years of age) were randomly chosen from oenology students who had completed two years of sensory instruction as part of Curtin University’s oenology program. The sensory instruction included wine fault identification and descriptors of wine faults including all of the wine descriptors used in this trial. Each respondent had previous experience on wine descriptive panels. To test respondent suitability, a subset of wines used in the sensory trial was presented under identical conditions. Wine samples of 50 mL were presented to respondents in random order, in clear, coded, covered, XL5 glasses at ambient room temperature (21 °C). All wines were expectorated after tasting and respondents were instructed to rinse their mouths by tasting and expectorating a 50 mL solution of rain water mixed with 5% pure lemon juice, followed by a 50 mL rinse of rain water in a forced rest between samples. The sensory trial was done in compliance with Curtin University’s Ethics Committee. The sensory attributes selected for aroma and taste were as reported for the sensory assessment of smoke tainted wines by Parker *et al.* [2]. Five descriptors of overall fruit, ash, solvent, medicinal and smoke aroma were recorded for each wine by smell alone and the seven descriptors of overall fruit, smoke, sourness, metallic, bitter, ashy aftertaste and drying were recorded by taste. The aroma scores for each wine were determined before the tasting of wines began. Respondents recorded sensory intensity of each attribute on a continuous 15 cm line with points marked “low” and “high” at 1.5 and 13.5 cm respectively. For multivariate analysis the phenols quantified in Kelly *et al.* [3] were screened by phenol wine concentration and their reported sensory thresholds [2,16,20,24].

### 3.5. Data Analysis

Data were analysed using a combination of univariate analysis of variance (ANOVA) and multivariate methods (principal component analysis (PCA) and partial least squares regression (PLSR)). Prior to these analyses, all the data were checked for satisfying the underlying assumptions. For the ANOVA, these involved testing homogeneity of the fuel treatment group variances and normality of each variate. For the PCA and PLSR, multivariate normality was evaluated using the radius test as outlined in GenStat for Windows V17 (VSN International, Hemel Hempstead, UK). The radius test showed all 12 sensory attribute variates examined had a multivariate normal distribution (*p* ≥ 0.16). Of the univariate normality tests, 2 out of 12 appeared to violate assumptions (0.02 < *p* < 0.05). However, even for these two cases, ANOVA following optimal transformation based on the Box-Cox procedure did not qualitatively alter the outcomes compared to the untransformed data suggesting robustness of the ANOVA to modest departures from normality. The ANOVA and PCA were performed in IBM SPSS Statistics 22 (IBM Corp, Armonk, NY, USA). A two-way ANOVA was carried out using the sensory attributes as response variables and vegetation sources of smoke (A), respondents (B), A × B, and the replicates as model factors. Mean separation was carried out using Fisher’s least significant difference. The PLSR analysis was performed using JMP V10 (SAS Institute Inc., Cary, NC, USA). The PCA was carried out on the correlation matrix. The PLSR was done on centred and scaled (*i.e.*, on standardised) variates with the wine chemistry (volatile and glycoconjugated phenols) and sensory attribute (aroma and taste) as the x-factors and y-responses variates, respectively. The optimum number of PLSR factors (which minimised root mean PRESS [predicted sums of squares error]) was determined with a leave-one-out cross validation. Contributions of the x-variables to the PLSR model were examined using a variable’s importance for projecting (VIP) the data into x-y score [25]. According to this criterion, a variable with a VIP score of 0.8 is minor. The final model presented included only x-variables with VIP ≥ 0.8.

## 4. Conclusions

The controlled application of smoke to Merlot grapevines, using vegetation fuel sources from the Margaret River region, has produced wines exhibiting differences in smoke taint aroma and flavour. This study differs from previous studies examining the sensory profile of smoke tainted wine as it has allowed comparisons not confounded by differences in variety and winemaking. The results show smoke taint is not only due to lignin derived phenols exhibiting negative aroma and taste descriptors but also from an elevation of phenols that mitigate the negative descriptors due to their softening effect. While a large number of phenols were found to be elevated in wines made from wildfire exposed vineyards [7] and may all contribute to changing the organoleptic profile, smoke taint was found here to be closely aligned to the concentrations of phenol, *m-*cresol and *p-*cresol glycoconjugates.
